# Exploring the Early Neolithic in the Arabian Gulf: A newly discovered 8,400–year-old stone-built architecture on Ghagha Island, United Arab Emirates

**DOI:** 10.1371/journal.pone.0326259

**Published:** 2025-06-25

**Authors:** Kevin Lidour, Noura Al Hameli, Rémy Crassard, Fabian D’Silva, Ahmed Al Haj

**Affiliations:** 1 Department of Culture and Tourism Abu Dhabi, Abu Dhabi, United Arab Emirates; 2 Historic Environment Department, CNRS, Archéorient UMR 5133, Maison de l’Orient et de la Méditerranée, Lyon, France; Tel Aviv university, ISRAEL

## Abstract

The site of GHG0088, with its two successive main phases of occupation, provides crucial data for re-evaluating our understanding of the Early Neolithic period (c. 6600–5400 cal. BCE) in the Arabian Gulf. The initial phase is marked by durable stone-built structures and evidence of domestic activities and funerary practices, presumably reflecting a settled lifestyle. The material culture includes a lithic industry, tools crafted from marine shells, and plaster vessels indicative of early pyrotechnological skills, while the absence of pottery challenges traditional views of Neolithic material assemblages in the Arabian Peninsula. Analysis of faunal remains indicates a subsistence strategy heavily reliant on marine resources, particularly fish, highlighting the exploitation of the neighbouring sea. While there is no evidence of agriculture or animal herding during that period, the rich coastal ecosystems likely ensured food security, reducing the need for residential mobility. The architectural remnants reveal patterns of continuity and adaptation across both phases. A significant layer of accumulated aeolian sand suggests a period of abandonment, potentially linked to the 8.2 ka BP climatic event. The subsequent reoccupation involved the adaptive reuse of the collapsed structures, transforming them into a temporary shelter for fishers, as suggested by numerous stone weights found. Additionally, the presence of shell beads underscores a renewed significance of marine resources during this second phase of occupation and suggests participation in extensive long-distance trade networks. These findings provide valuable new perspectives on the early stages of the Neolithic period in the Arabian Gulf. Comparisons with other contemporary sites offer a foundation for redefining the Early Arabian Neolithic and its timeline in this region.

## Introduction

**Fig 1:**
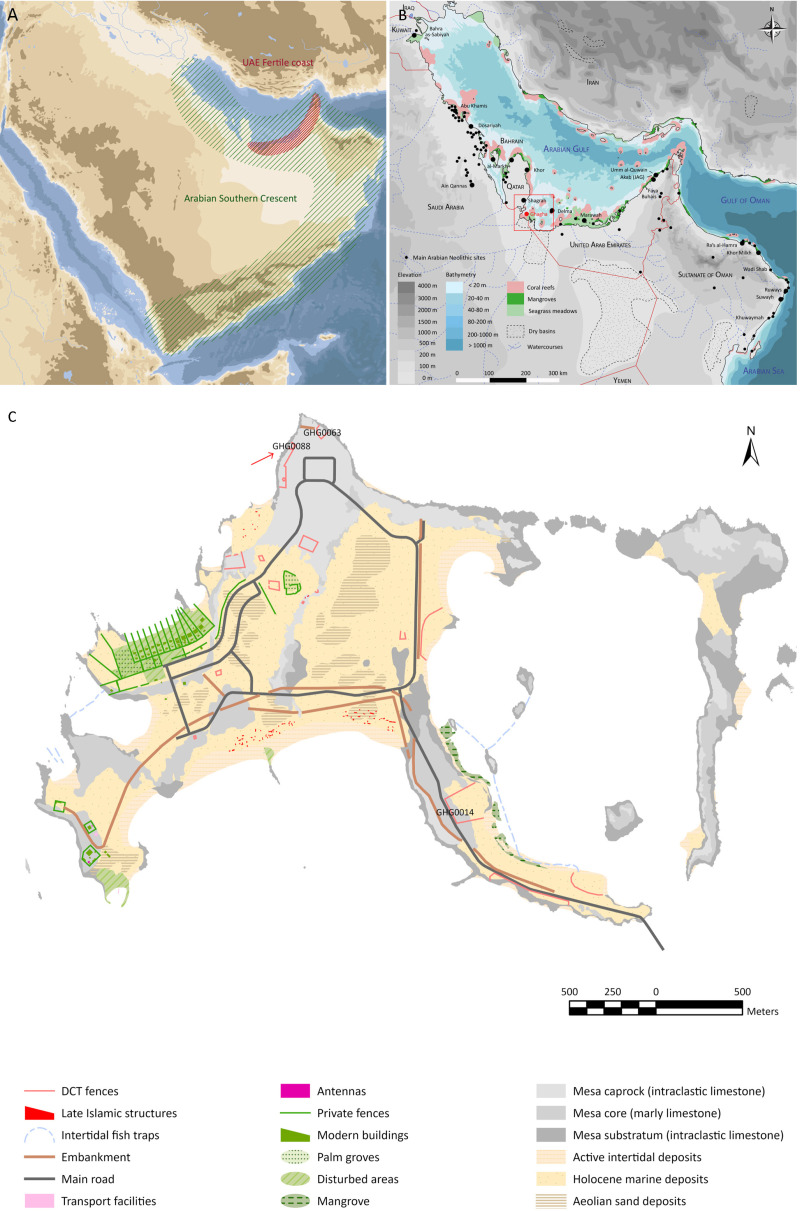
A. map of the Arabian Peninsula showing the location of the Arabian ‘Southern Crescent’ and the UAE ‘Fertile Coast’; B. Map of the Arabian Gulf and northern Sultanate of Oman indicating the main Neolithic sites; C. Map of Ghagha Island showing the locations of the main Early Arabian Neolithic sites. All maps were created by K. Lidour using ArcGIS Pro 3.2.0 and Adobe Illustrator 28.0, and are published under the CC BY 4.0 license.

The Arabian Peninsula, characterised by its arid and semi-arid climatic conditions throughout the Holocene, has long been a region where human groups have had to adapt to challenging environments. These adaptations often led to migrations towards ecological refuges, particularly tropical coastal ecosystems rich in biomass and biodiversity, such as mangroves, seagrass meadows, and coral reefs [1,2]. The coasts of South-East Arabia have been recently conceptualised as a ’Southern Crescent’ by Rose [3], and as the ‘Fertile coast’ by researchers from the Department of Culture and Tourism of Abu Dhabi [4] (Fig 1A) as a means of counterbalancing the cultural centralism of the Fertile Crescent in Neolithic studies, especially in South-West Asia. These concepts highlight the unique coastal adaptations of South-East Arabia, which contrast with the agrarian-based neolithisation processes in the Levant. In South-East Arabia, the Neolithic is conventionally defined as the cultural phase preceding the Bronze Age, which begins around 3300 BCE. The chronological scope and definition of the Arabian Neolithic remain subjects of debate, particularly concerning its earlier phase, which still lacks sufficient documentation.

During the regional Neolithic period (c. 6600–3300 BCE), numerous coastal settlement sites, from Kuwait to the Sultanate of Oman, show a strong reliance on marine resources, sometimes also evidenced by the presence of shell middens near productive coastal ecosystems (Fig 1B) – in particular lagoonal mangroves. Coastal ecosystems present in the region have been particularly pivotal in the construction of a unique path within the neolithisation process [5] associating marine resource exploitation, pastoralism, and hunting [6]. Sustainable fishing-based economies are expected to allow for perennial coastal settlement [2], possibly fostering the early emergence of stone-built architecture in some areas, such as on Marawah and Ghagha islands (Emirate of Abu Dhabi, United Arab Emirates).

The present study investigates the recently discovered Neolithic site GHG0088 on Ghagha Island (Fig 1C), contributing to the broader understanding of the Abu Dhabi Western Region’s archaeological landscape. Conducted by the Historic Environment section of the Department of Culture and Tourism (DCT) of Abu Dhabi, this research is part of a larger project aimed at documenting Neolithic sites on Ghagha Island, spanning from 2019 to 2024. The excavation builds on previous work at sites GHG0014 and GHG0063. The initial phase of occupation at GHG0088 dates back to around 6400–6200 BCE and is linked to one of the earliest stone constructions in the region (Early Arabian Neolithic 1). Further, albeit sparse, activity on the site has been dated to around 6000–5800 BCE. The subsequent major phase of occupation is dated to around 5700–5400 BCE (Early Arabian Neolithic 2). Associated remnants of stone architecture have been uncovered during this phase as well, suggesting their use as a shelter by a small fishing community but likely for a relatively short period. The artefacts and animal remains recovered from the site offer an opportunity to examine and define more precisely the Early Neolithic in the Arabian Gulf.

### Geographical setting

Ghagha Island is situated in the westernmost part of the Al Dhafrah region, approximately 300 km west of Abu Dhabi Island, United Arab Emirates. The island is connected to the Ras Ghumais peninsula by a bridge over a 4 km wide stretch of water. To the north, it is about 15 km from Ras Abu Gamys in Saudi Arabia, to the south of the Qatar Peninsula.

The island is more of an archipelago, composed of several limestone mesas surrounded by Holocene marine deposits [7] (Fig 1C). The main island covers about 3 km^2^. It is separated from the smaller Qassar Ras Dhawi (0.4 km^2^) to the east by a shallow lagoon-like depression. The elevation is very low, with the highest areas not exceeding 15 m a.s.l. The landscape is dry, with no available surface freshwater. There is minimal seasonal rainfall (< 50 mm annually), resulting in limited grass cover and only salt-tolerant vegetation on the upper beach areas and in the vicinity of inland evaporite basins (*sabkhat*). A few mangrove trees (*Avicennia marina*) concentrate in the shallow flooded depression separating Ghagha Island and Qassar Ras Dhawi. However, recent developments such as a water catchment system and a few wells have enabled small-scale cultivation of date palms (*Phoenix dactylifera*) on the island. The islands have not been systematically assessed for their animal and plant populations, but the limited human activity has helped preserve natural habitats and the wildlife that depends on them. There are reports of several pairs of ospreys (*Pandion haliaetus*) nesting on the island, as well as communities of dugong (*Dugong dugon*) grazing in the surrounding waters. Additionally, the nearby island of Khirdal is home to a significant colony of the endangered Socotra cormorant (*Phalacrocorax nigrogularis*). Traces of extensive presence of cormorants have been observed as well in the northern part of the Qassar Ras Dhawi. Historical records indicate that seabird guano collection was locally exploited in the past, with the intention to export it for trade [8].

Nowadays, Ghagha Island holds no natural sources of freshwater and relies on desalination plants and truck deliveries. In the recent past, freshwater deliveries were also ensured by boat shipments from islands where this resource was abundant, such as Delma Island [9]. Ethnographic sources reveal that freshwater was historically sourced from shallow wells, artesian springs, and even resurgences at the bottom of the sea. Brackish water was traditionally mixed with goat milk to enhance drinkability [10]. Rainwater harvesting was also documented in the recent past, with water runoff being redirected by ditches and retaining walls into collection basins. These features are sparsely observed across the landscape of the Abu Dhabi Western Region.

In the recent past, the island was predominantly inhabited by Al Qubaisat families, a section of the Bani Yas tribe. Late-Islamic villages composed of mosques and permanent stone houses are mainly found in the north-western and southern parts of the islands. Their primary activities were focused on pearl diving and local fishing, including using intertidal traps. According to oral history accounts, Ghagha Island was also utilised as a quarantine base, where sick individuals were isolated with enough provisions for subsistence, thus preventing contact with other coastal communities. Navigation in the surrounding waters is quite perilous due to shallow depths, numerous reefs, and sand bars. Local islanders have constructed numerous small stone cairns to assist in guiding boats. Nowadays, modern houses and chalets have been constructed in the western part of the island where Al Qubaisat families and occasional visitors reside seasonally, such as for weekends and holidays.

### Archaeological setting

The island of Ghagha was initially explored by the Abu Dhabi Islands Archaeological Survey (ADIAS) project from 1993–1995 [11,12]. This first survey revealed evidence of Late Stone Age-Neolithic presence on the island, although not dated precisely at that time. This was characterised by the presence of lithic artefacts associated with the obsolete ‘Arabian Bifacial Tradition’ (ABT) [13] previously dated from the 5^th^ millennium BCE (unpublished report from J. Czastka, [11]). Additionally, traces of occupation spanning from the Bronze Age to the Middle-Late Islamic periods were also discovered. In November 2012, the island was further investigated by the Archaeological Prospection Services of the University of Southampton (APSS) and the Maritime Archaeology Stewardship Trust (MAST) in collaboration with the Department of Culture and Tourism (DCT) of Abu Dhabi [14]. The subsequent fieldwork included extensive archaeological reconnaissance on both land and intertidal areas, as well as geophysical surveys using Ground Penetrating Radar (GPR) and magnetometry in specific areas of the island where large stone cairns and Late Pre-Islamic architectures concentrate.

DCT Abu Dhabi conducted additional surveys between 2018 and 2021, leading to the reexamination of some of the previously identified large stone cairns. Excavations at GHG0014 from 2019 to 2021 revealed a stone-built architecture dating back to 6600–6500 BCE, associated with ancient fishing activities [4]. So far, it consists of the oldest stone-built architecture of the Arabian Neolithic in the Gulf region. This site also yielded a lithic assemblage and the earliest evidence of plaster vessel making in the region. Excavations continued from 2021 to 2024 at GHG0063, highlighting a settlement dedicated to the production of plaster vessels dating back to 6400–6200 BCE (inedit data). Although no distinctive stone-built architecture was identified, GHG0063 has provided a large, diversified lithic industry, including many projectile points.

**Fig 2:**
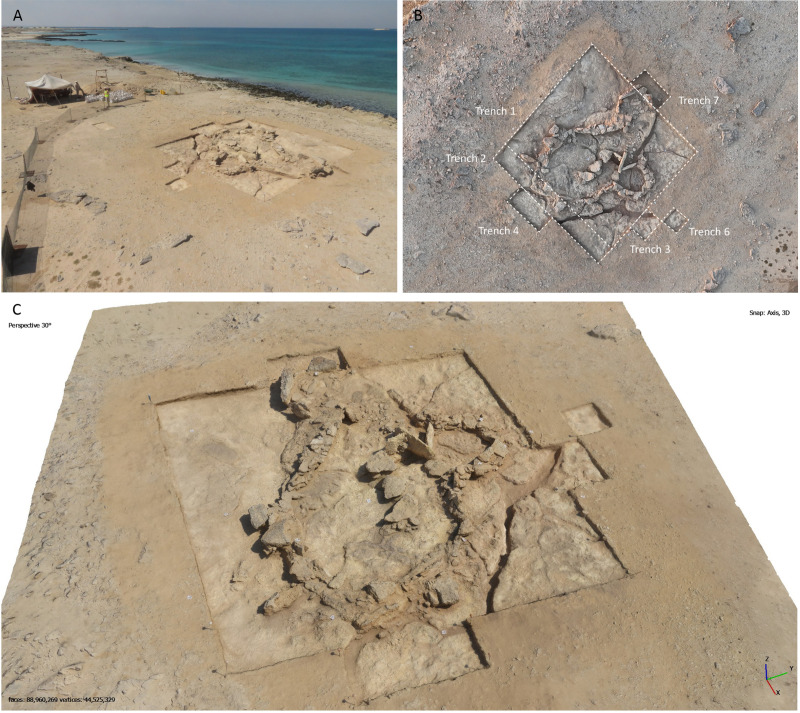
A. Oblique aerial photograph of the GHG0088 site at the end of excavation, with the Phase 1 structures fully exposed; B. Zenithal photograph (DJI Mini 4 Pro) of the GHG0088 site (Phase 1), showing the location of the excavation trenches; C. Oblique view of a 3D model of GHG0088 (Phase 1) (generated using Agisoft Metashape Professional 2.1.0).

During a survey conducted in November 2023, a chert tanged and barbed projectile point (SF0001) was found at GHG0088 (WGS84(DMS) 24°25’22.52388” N; 51°32’42.61848” E), a 7 m wide mound located about 180 m to the south-west of GHG0063. This site lies on the edge of a caprock limestone mesa (c. 7.2 m a.s.l.) facing west towards the sea, about 25 metres distant. A narrower platform (c. 3.5 m a.s.l.) of marly limestone, around 15 metres in width, lies between the upper platform, where the site is located, and the sea (Fig 2A). The discovery of a chert projectile point indicated a potential Neolithic occupation. Subsequently, the full excavation of GHG0088 took place in February-March 2024.

## Materials and methods

An initial area (7x8 m) was excavated where buried architectural structures were expected, thus encompassing the entire mound. Further extensions were added to the initial excavation area to capture the full extent, as well as verify the presence of any further activity around the mound (Fig 2B). An additional trench (1x8 m) was excavated to complete the general plan of the structure, resulting in a unified excavation area of 64 m². Additional small excavation areas added a further 9 m².

Excavations were carried out using the single-context recording (SCR) system. Each context was assigned a unique identifier – SU for deposits and fills, [SU] for constructions –, described on a standardised context sheet, photographed and georeferenced as an individual entity. The georeferencing was carried out using a Leica GNSS RTK rover (GS16). Additionally, various series of photographs of the site were taken (including with a drone, DJI Mini 4 Pro) during the excavation process to produce 3D models and orthomosaics using Agisoft Metashape Professional 2.1.0 (Fig 2C; S1 File).

The stratigraphic diagram (S1 Fig) was generated using Le Stratifiant 0.3.7 software developed by Desachy [15]. It consists of a set of program modules written in VBA language and used as a series of macro commands within Microsoft Excel, whose graphic functions are employed to plot the diagram. The ortho-linear diagram produced deviates from the traditional Harris matrix [16] by consistently representing anteroposterior relationships with unbroken vertical lines and synchronic relationships with horizontal lines, enhancing clarity and reducing interpretative errors in large diagrams. While it requires multiple labels for a single stratigraphic unit (SU) with several connections, it maintains the integrity of the Harris matrix principles, such as excluding redundant relationships, to preserve a concise stratigraphic chronology.

All the sediment excavated was dry sieved using a 3 mm mesh. Artefacts and faunal remains were fully collected and bagged. Bulk samples were taken for flotation from the richest soils, particularly those yielding ash and charcoal. Samples were typically 10 litres in volume; approximately the volume of a bucket. The amount of material excavated came to a total of almost 20,000 litres (20 m^3^). Artefacts and other significant discoveries have been assigned Special Finds (SF#) numbering. Bulk samples (BS#) and charcoal (CHARC#) were recorded separately.

## Results

### Phasing

#### Phase 1 – Early Arabian Neolithic 1 (‘Ghagha Phase’).

**Fig 3:**
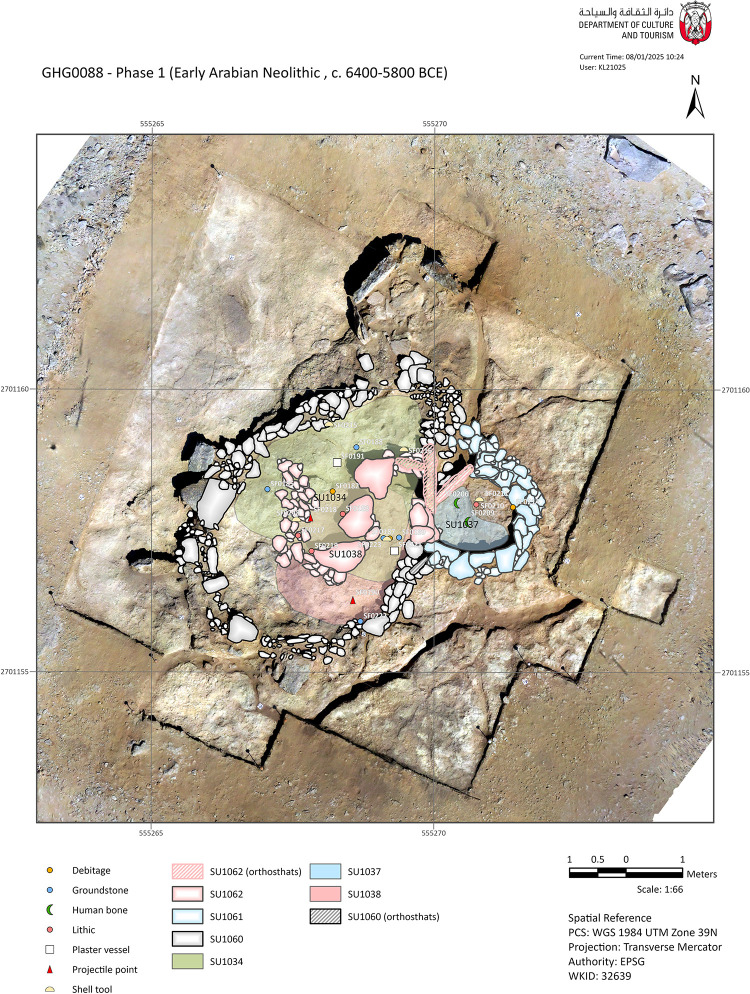
Plan of the Phase 1 structures, main deposits, and findings (CAD: K. Lidour, created using ArcGIS Pro 3.2.0).

**Fig 4:**
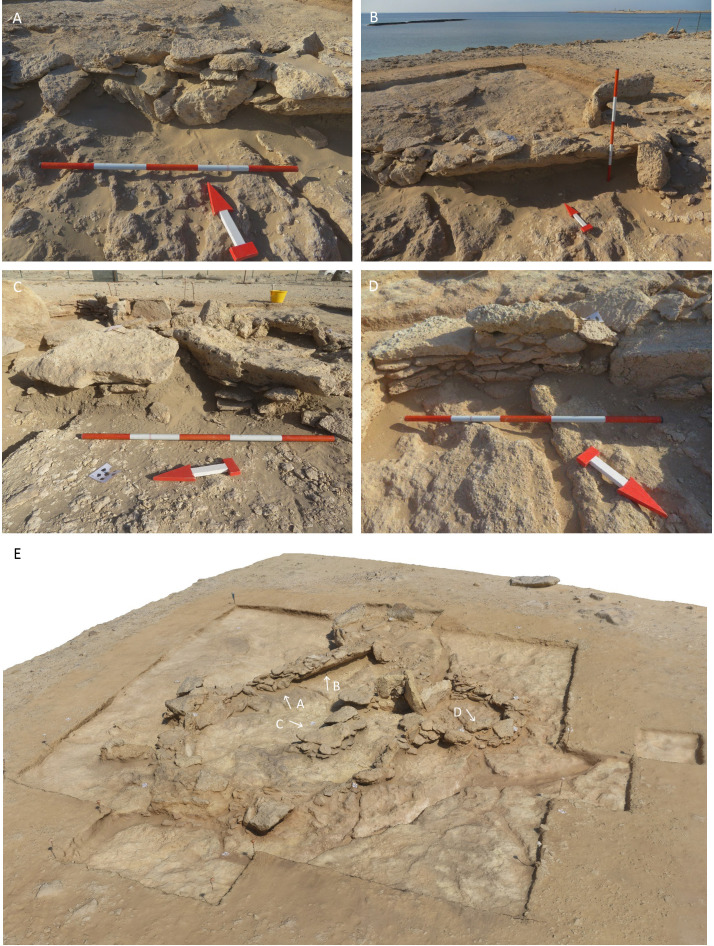
Selection of detailed views of architectural elements from the Phase 1 structures of GHG0088.

Stratigraphically, no deposits have been found that predate the construction of the main cell wall [SU1060]. Wall [SU1060] has a perimeter of 15.5 m, with only a 1 m wide gap where a further wall [SU1061] was constructed (Fig 3). The inner space measures approximately 4.7x3.3 m (11.2 m^2^). The wall’s thickness ranges from 25 to 55 cm, with a maximum preserved height of about 35 cm, often comprising one to two courses of stones. In some sections of the wall, particularly in its western extent, up to five courses of stones are preserved. Stones up to 40 cm in length were occasionally placed vertically or angled to fill gaps in the uneven bedrock. Smaller flat stones measuring 10–15 cm were also used to level the surface for laying the lower courses of the wall (Fig 4A). Large stones (30–50 cm) are arranged in pairs on the wall’s top – which it is still visible in the southwest extent of [SU1060]. The stones used for construction seem locally sourced, being composed of fine-grained limestone or friable marine conglomerate with fossilised shells – they typically measure up to 30x50x15 cm. No traces of mortar were observed.

The northeastern section of [SU1060] seems to have been dismantled. In this area, instead of the usual stone courses, small flat stones [SU1025] were placed directly on the bedrock to level its surface. This suggests that a new entrance might have been opened there after the wall [SU1061] has obstructed the entrance associated with the earlier structure. We also should consider that [SU1060] might be mainly preserved beneath the threshold stone – with only the first foundation courses remaining. This means that the entrance might not be visible.

In the main cell, the earliest deposit identified is SU1038. It is an oval-shaped lens of dark grey ashy sand concentrated in the southeastern part of the cell. This deposit did not yield a substantial number of finds. It includes a small pink quartz pebble used as a hammerstone (SF0222), a shell tool (SF0223), a cluster of five plaster vessel sherds with red painted decoration (SF0221), and a fragment of a retouched blade (SF0218) – possibly a projectile point fragment. SU1034 is another sandy lens that overlies SU1038. It comprises loose greenish sand (containing finely degraded marly limestone) and includes faunal remains and a few artefacts such as lithic implements and shell tools. This deposit is sealed by rubble layers (SU1017 and SU1018), which have very few associated artefacts likely derived from disturbances of the surface of the occupational floor. These artefacts include the tang of a projectile point (SF0192), the tip of another barbed and tanged point (SF0190), and fragments of grinding stones (SF0187, SF0188, and SF0189).

In the main cell, SU1034 is located below a set of internal structures grouped as [SU1062]. [SU1062] contains the remains of a potential wall, aligned north to south for 1.9 m. It then begins to curve to the east at its southern extent. It seems to continue further under one of three large stones lying flat (Fig 4C). One of these large stones directly supports a series of three large orthostats that block the doorway between the main and the small cell. The purpose of the potential wall associated with [SU1062] is not clearly understood, but it appears to be related to a subdivision of the internal area of the main cell.

Wall [SU1061] defines a smaller enclosed space, with a perimeter of 2.2 m and an area of approximately 2 m^2^. The preservation is overall good, with up to eight courses remaining and a height of approximately 25–35 cm (Fig 4D). The function of this confined area remains uncertain; however, its designation for domestic use is questionable due to the limited internal space – although charred materials were collected and radiocarbon-dated from SU1037. While it appears that [SU1061] was constructed adjacent to, and thus after, [SU1060], there is no clear evidence of a significant chronological gap between the two walls, despite their differing states of preservation. However, the conditions of stratification in the open-air during this period remain uncertain. Deposits of aeolian sand are likely to have been preserved only within the inner structures, while external deposits may have been blown away. The fact that both [SU1060] and [SU1061] are built directly on the bedrock does not provide strong evidence for their contemporaneity.

It is important to note that human remains, including a long bone diaphysis and a fragment of a flat bone, likely a coxal bone in very poor condition, were discovered in the small cell. The remains were found at the interface of SU1024 (a loose aeolian sand deposit) and SU1035 (a stack of stone overlying SU1037). A physical anthropologist (FD) meticulously sifted through SU1024, SU1035, and SU1037 (totalling c. 400 litres of sediment) using a 1 mm mesh hand sieve but did not find any additional bone fragments. It thus rules out the possibility of a primary burial originally installed in the small cell, before being disturbed. We can confidently conclude that the two human bones consist of a secondary burial and were originally brought from another location.

**Fig 5:**
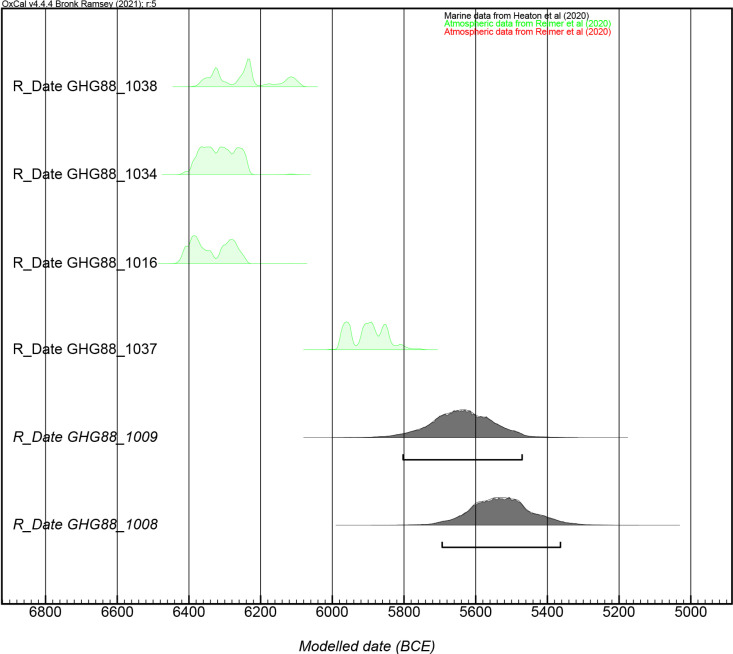
Multiple radiocarbon dates from Neolithic contexts at GHG0088 (generated using OxCal 4.4.4).

Phase 1a at GHG0088 has been dated to around 6400–6200 BCE based on radiocarbon dating of charcoal samples from SU1016, SU1034, and SU1038 (Table 1; Fig 5). This means that Phase 1a overlaps with the occupation of GHG0063. Both sites exhibit similarities but slight differences in their organisation and material culture.

**Table 1:** Radiocarbon dating from GHG0088. 710689 and 710688 calibrated with Marine20 curve, DeltaR = 46 + /- 53. Processed by Beta Analytic (USA). Atmospheric data from Reimer et al. (2020); Marine data from Heaton et al. (2020); OxCal v4.4.4 Bronk Ramsey (2021).

Beta No.	SU	Material	Conventional age	Calendar calibration	‰ δ13C	‰ δ18O
697765	1038	Charred material	7380 + /− 30 BP	(73.7%) 6376–6213 cal BCE (8325–8162 cal BP) (19.2%) 6161–6088 cal BCE (8110–8037 cal BP) (2.4%) 6187–6167 cal BCE (8136–8116 cal BP)	−12	–
697766	1034	Charred material	7440 + /− 30 BP	(95.4%) 6392–6237 cal BCE (8341–8196 cal BP)	−10.8	–
697767	1016	Charred material	7480 + /− 30 BP	(53.2%) 6422–6331 cal BCE (8371–8280 cal BP) (42.2%) 6320–6246 cal BCE (8269–8195 cal BP)	−10.5	–
702451	1037	Charred material	7010 + /− 30 BP	(95.4%) 5985–5803 cal BCE (7934–7752 cal BP)	−10.8	–
710689	1009	Marine shell (*Marcia* sp.)	7650 + /− 30 BP	(95.4%) 6093–5732 cal BC (8042–7681 cal BP)	0.88	1.46
710688	1008	Marine shell (*Marcia* sp.)	7230 + /− 30 BP	(95.4%) 5693–5363 cal BC (7642–7312 cal BP)	3.86	2.1
702450	1055	Organic sediment	500 + /− 30 BP	(95.4%) 1399–1450 cal CE (551–500 cal BP)	−10.9	–

Phase 1b corresponds to the occupation of SU1037, the earliest deposit associated with the small cell, which has been radiocarbon-dated to approximately 6000–5800 BCE. Consequently, the burial located higher in the stratigraphy cannot be associated with Phase 1a, as there is a chronological gap of at least 200 years. Furthermore, it cannot be later than the date obtained for SU1009, which is 6093–5732 cal. BCE (Beta 710689). However, there is some uncertainty regarding the marine reservoir effect, as this date was derived from a shell valve (*Marcia* sp.) in the absence of charcoal. This dating further supports the evidence of a brief occupation at the site during the first half of the 6^th^ millennium BCE.

#### Phase 2 – Early Arabian Neolithic 2 (‘Marawah Phase’).

SU1009 and SU1016 constitute a thick sand infill within the main cell that was comparable to a massive sand mound during the excavations, roughly oval-shaped and measuring approximately 5x3.2 m (40 cm max. depth). Artefacts from SU1009 are concentrated in the upper part of the stratum and are comparable to those associated with SU1004. The occupation floor associated with the second phase has likely been disturbed and fragmented into SU1004, SU1008, and the uppermost part of SU1009. In contrast, SU1016 is an almost sterile accumulation of aeolian sand, representing a distinctive hiatus between the Phase 1 and the Phase 2.

**Fig 6:**
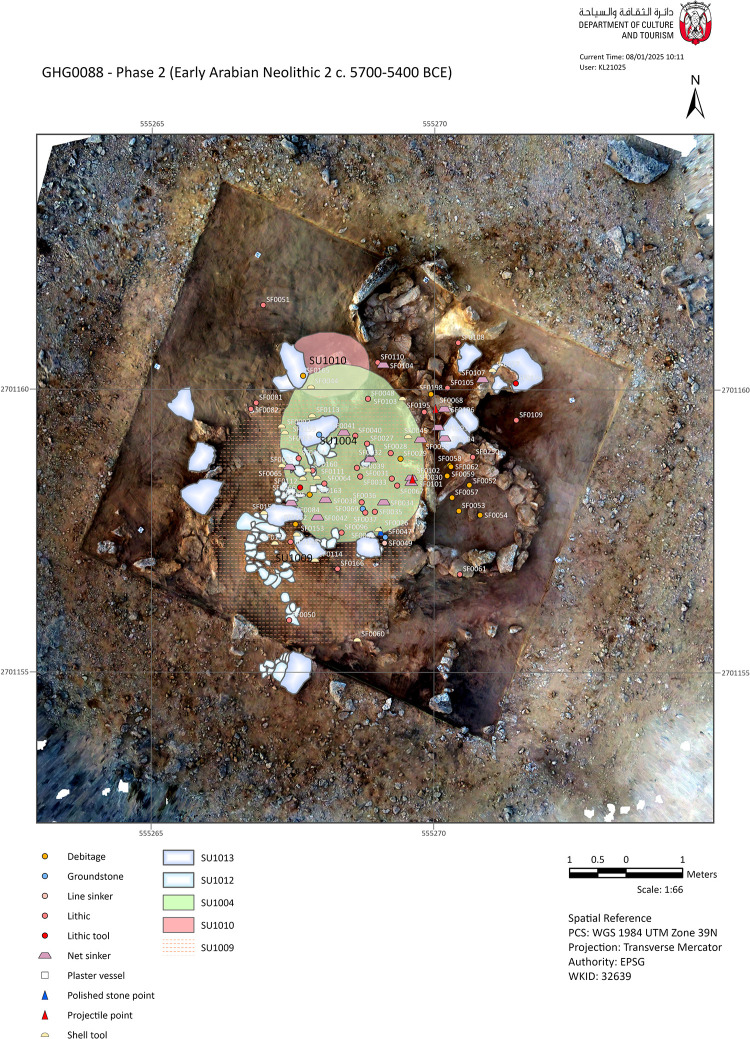
Plan of the Phase 2 structures, main deposits, and findings (CAD: K. Lidour, created using ArcGIS Pro 3.2.0).

**Fig 7:**
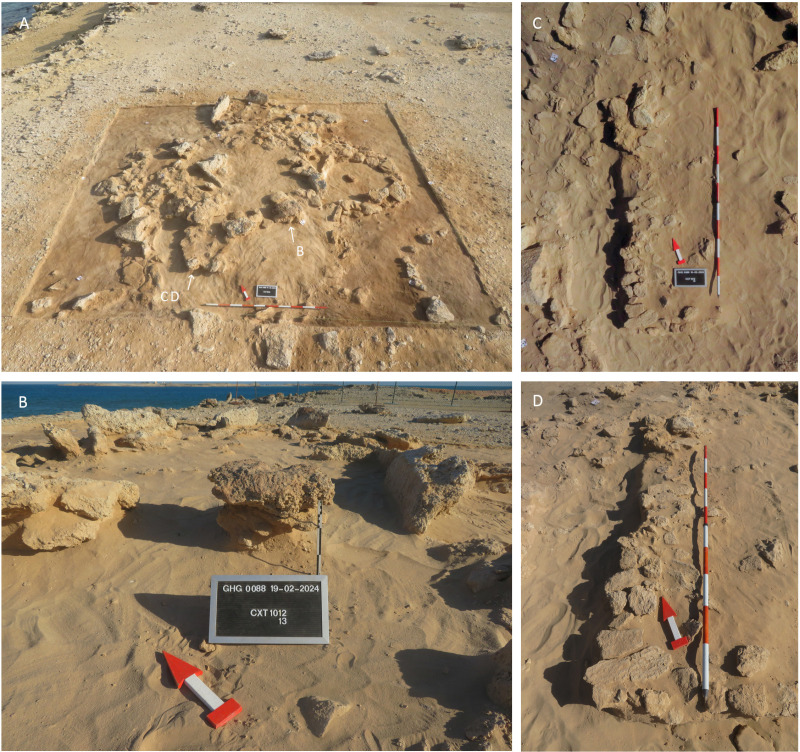
Selection of detailed views of architectural elements from the Phase 2 structures of GHG0088.

[SU1012] is a paved feature approximately 2.75 m in length and about 50 cm wide, fragmented into several small patches that have been set atop SU1009 (Figs 6 and 7C–D). It consists of stone tile flooring likely installed to consolidate the top of SU1009, which is a soft infill of aeolian sand. This tile flooring does not appear to have been used as a circulation floor but rather as a means of consolidation for building a wall. A further series of large (60–75 cm) flat stones [SU1013] (Fig 7B) were installed atop [SU1012], believed to be remnants of an architecture, probably made with vegetal materials in some parts. SU1014 and the associated cut SU1015 are interpreted as the remains of a posthole that may have been part of the architecture, probably installed to support a roof. The spatial distribution of artefacts clearly delineates an oval-shaped area measuring 3.5x3 m at the top of the sand accumulation SU1009.

The main occupation has been identified in association with SU1004, SU1008, and the top surface of SU1009. While SU1008 and SU1009 were deposits of loose sand, SU1004 is more compacted sand, lying directly beneath SU1003 (compacted silty sand) near the surface. SU1004 has yielded a greater concentration of artefacts, including knapped lithics, marine shell beads and tools, as well as numerous stone fishing weights. Further weights were found in the surrounding rubble deposits. A hearth (SU1010) was recorded directly northwest of SU1004. The hearth measures about 1 metre in diameter and consists of a spread of ashy sand at the surface. It rests directly on top of wall [SU1060]. The associated finds include a retouched chert flake (SF0126), and a shell tool (SF0127).

In the absence of charcoal, a marine shell (*Marcia* sp.) from SU1008 was radiocarbon-dated to 5693–5363 cal. BCE (Beta 710688). The material from this context closely aligns with that from SU1004, suggesting that both samples originate from the same episode of occupation. Consequently, Phase 2 is dated to approximately 5700–5400 BCE.

#### Phase 3 – Deflated deposits and Islamic period.

**Fig 8:**
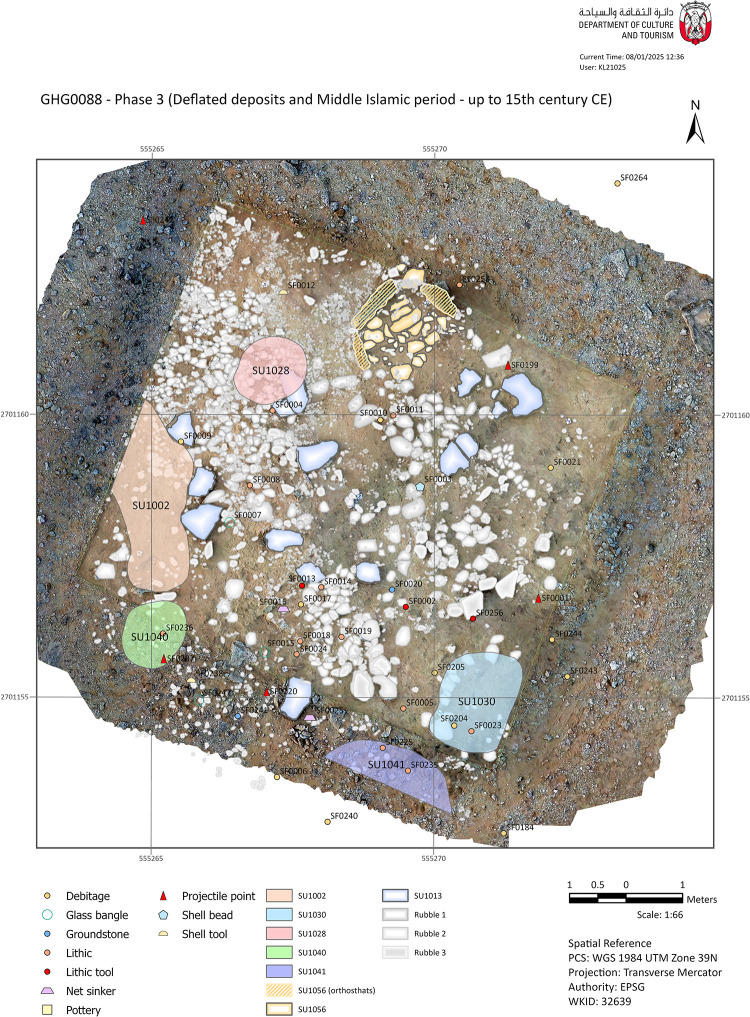
Plan of the Phase 3 structures, main deposits, and findings (CAD: K. Lidour, created using ArcGIS Pro 3.2.0).

[SU1056] is another architectural unit located in the northern part of the main excavation area. It is stratigraphically associated with Phase 3, but no dating evidence could be retrieved (Fig 8). It comprised three large orthostats, roughly delineating a square paved area of 1.2x1 m in width. The paving is composed of limestone slabs up to 70 cm long that lie flat on pure sand and appear to level out the natural disparities in the bedrock. The function of this structure remains completely unknown.

**Fig 9:**
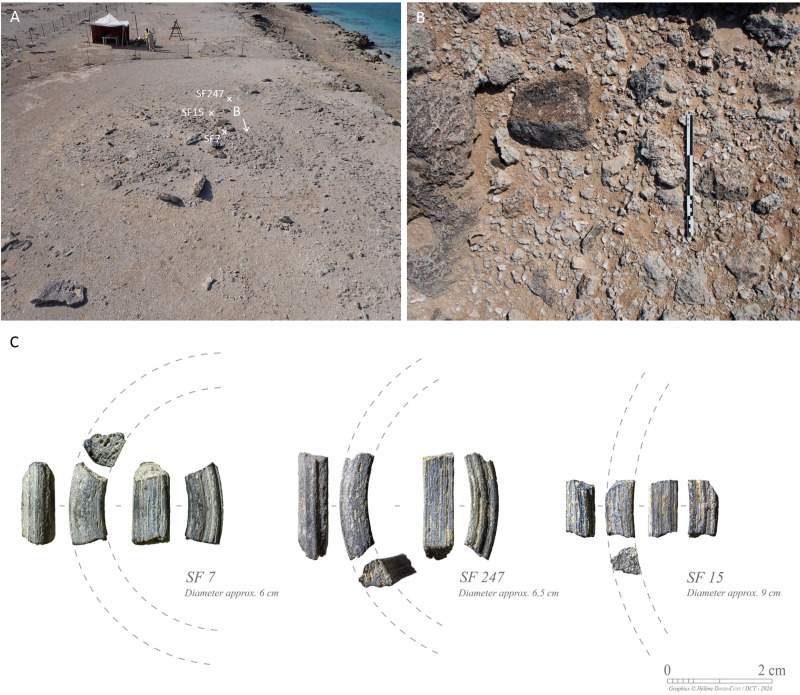
A. Oblique aerial photograph of the GHG0088 site at the beginning of excavation, showing the location of the glass bracelet fragments and the concentration of murex shells; B. Detail of the concentration of fragmentary murex shells associated with Phase 3. C. Glass bracelet fragments recovered from Phase 3 (Photos: H. David-Cuny).

The latest phase of occupation includes the uppermost stratigraphic layers (SU1001, SU1002, and SU1003). Finds unequivocally associated with the Neolithic occupation comprise five projectile points (SF0001, SF0199, SF0220, SF0237, SF0242). It is crucial to acknowledge that these points were found in areas with very shallow stratigraphy and where bedrock is quite close to the surface. These areas have been more susceptible to deflation. As a result, they could be linked with any of the Neolithic phases (Phase 1 or 2). However, the discovery of glass bangle fragments (SF0007, SF0015, SF0247) also indicates later disturbance at the surface of the site. These fragments vary in size and appear to belong to bangles or bracelets of different diameters, yet all exhibit a similar triangular section and a ribbed decoration on one of their sides. Islamic glass bangles are thought to have been largely diffused in the Arabian Gulf from the 13^th^ until the 18^th^ century CE (Fig 9C) [17,18].

Only a concentration of murex shells (*Hexaplex kuesterianus*) (SU1002), can be strictly assigned to the Islamic period (Fig 9B). This deposit represents the uppermost context in the stratigraphic sequence, lying above the surface rocks (SU1001). Similar large spreads of murex shells are observed all along the edge of the cliff both to the north and the south of the site, including at GHG0086 and GHG0087. They are occasionally found with scatters of Julfar ware potsherds, indicating a Middle-Late Islamic dating (12^th^–20^th^ centuries CE).

Further remnants of late human activities on site extend around the mound, mostly in the southern extent of the excavation area. Some of these deposits are directly on the bedrock and have very few findings, such as burnt bird bones and almost no artefacts. Notably, clusters of chert flakes and a bladelet have been discovered in association with SU1030. A charcoal fragment from SU1055 has provided a radiocarbon date of 1399–1450 CE (Table 1). It thus confirms the typological dating previously suggested.

The site’s top surface has also yielded cormorant bones, bird feathers, and modern fish remains, indicating reoccupation by wildlife. This is particularly evident from ospreys, which may have used the archaeological mound as a perching and resting spot. This is supported by the presence of fresh remains of tripodfish (*Triacanthus biaculeatus*), a species frequently preyed upon by ospreys in the region [19].

### Animal economy

The faunal assemblage totally lacks land animal remains. Consumed fauna includes only marine molluscs, fish, and seabirds in lesser quantities. The majority of remains come from surface deposits. SU1002 is linked to the exploitation of murex during the Middle Islamic period (Phase 3) – it mainly consists of *H. kuesterianus* shells. SU1001 and SU1003 also have *H. kuesterianus* remains, along with other species like *Asaphis violascens*, *Priotrochus obscurus*, and *Pinctada radiata*. This indicates the exploitation of intertidal and shallow subtidal rocks, aligning with the characteristics of the local marine ecosystem. The ashy deposits surrounding the stone structures have yielded very few seashells and almost no fish remains. The sole seashells collected are small gastropod shells such as *Drupella margariticola* and *P. obscurus*. Contexts located in the southern extent of the excavation area have produced numerous fragments of burnt bird bones.

Contexts linked to Phase 2 yield a considerable collection of faunal remains, primarily marine shells. The species represented include *A. violascens*, *P. radiata*, *P. obscurus*, *Lunella coronata*, *Tylothais savignyi*, *D. margariticola*, and some *Marcia* sp., with infrequent shell fragments of *Barbatia* sp. It evidences that shell collection was primarily focused on the exploitation of intertidal and shallow subtidal rocks and, in lesser importance, on coarse sand and gravel flats. Notably, the upper surface of SU1009 has revealed numerous non-edible seashells that do not appear to have been repurposed as personal ornaments; this includes many *Mitrella blanda*, *Nassarius* spp., and small cerithids. The hearth in SU1010 has yielded a small cluster of burnt and cracked *H. kuesterianus* shells. In contrast, ichthyofaunal remains are scarce and poorly preserved. A limited number of otoliths from grouper (*Epinephelus coioides*) and vertebrae from small sawfish (Pristidae) were identified in Phase 2. They likely highlight the exploitation of surrounding subtidal reefs and seagrass areas. Several fishing weights were retrieved from Phase 2 contexts, highlighting the use of fishing nets by the ancient inhabitants. Fishing nets used during the Neolithic in South-East Arabia were probably small beach seines, allowing for the non-selective capture of coastal fish [2].

Deposits from Phase 1a have produced a richer and more diverse fish assemblage: in SU1018, remains attributable to groupers, emperors (*Lethrinus nebulosus*), and potentially flatheads (*Platycephalus indicus*) were found. Occupational layers SU1034, SU1038, and SU1050 have also yielded fish remains, including jawbones and otoliths from groupers and flounders (*Pseudorhombus arsius*). The recurrence of groupers, flatheads and flounders in the fish assemblage might suggest that a part of the fishing effort included the use of spears or harpoons in shallow waters. Surprisingly, marine shells are virtually absent from contexts associated with Phase 1, which contain only very sparse and small fragments of *A. violascens*, *P. obscurus*, and *D. margariticola*.

### Material culture

#### Lithic industry.

The lithic analysis focuses on the definitive assemblage from GHG0088, which consists of 115 artefacts, each assigned a unique SF number and with many specimens (46%) carefully plotted in situ.

The raw materials used for lithic production at GHG0088 are notably high in quality, including fine-grained chert and jasper-like flints (58.8%) characterised by their bright colours and excellent knappability. Other raw material used include quartzite (22.8%) and quartz (18.4%). Potential sources of raw materials, including brownish flint and quartzite, have been identified in several locations on the nearby mainland, particularly on the gravel-covered Miocene plateaus of the Sabkha Matti region. Some of the raw materials, particularly the jasper-like flints, might have been obtained from more distant sources.

Despite the high quality of raw materials, the overall paucity of the lithic assemblage at the site raises questions about the nature of its occupation and activity – while the use of shell tools is also expected to have compensated for the lack of lithic resources. Nevertheless, this scarcity will need to be critically evaluated in terms of taphonomic factors, such as post-depositional processes that might have contributed to the loss of lithic materials. The limited number of lithics could also reflect an actual absence of significant knapping activity on site.

**Fig 10:**
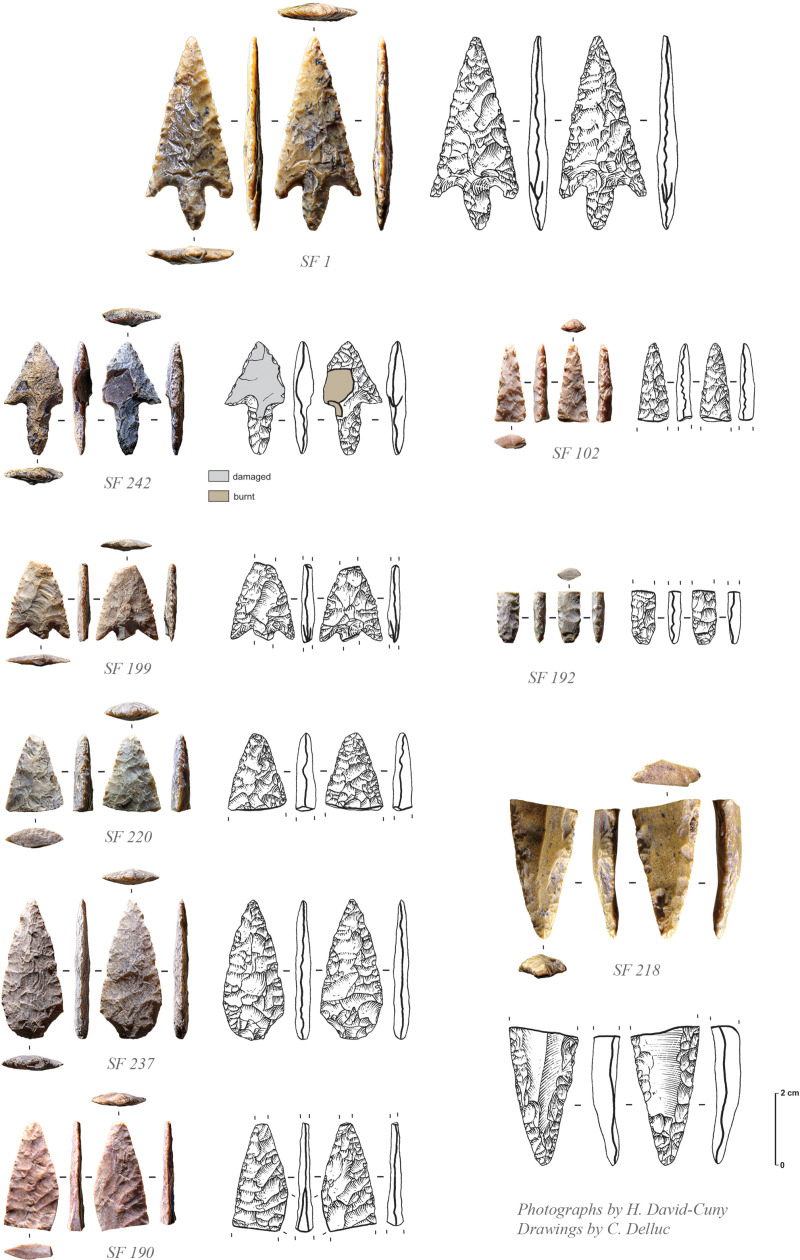
Projectile points recovered from GHG0088 (Photos: H. David-Cuny; Drawings: C. Delluc).

**Fig 11:**
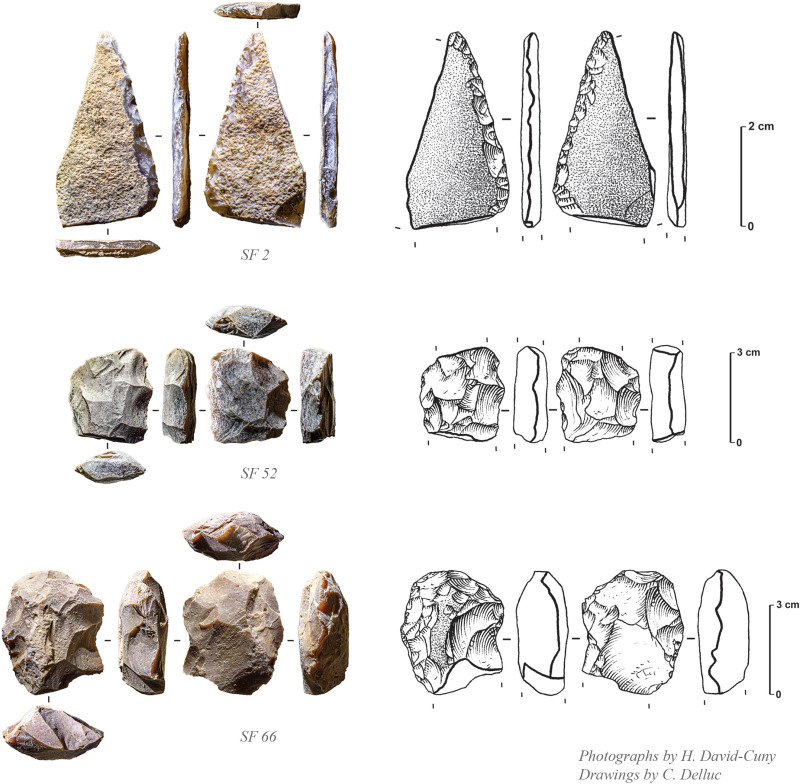
Tile knife (SF2) and bifacial pieces (SF52 and SF56) from GHG0088 (Photos: H. David-Cuny; Drawings: C. Delluc).

**Fig 12:**
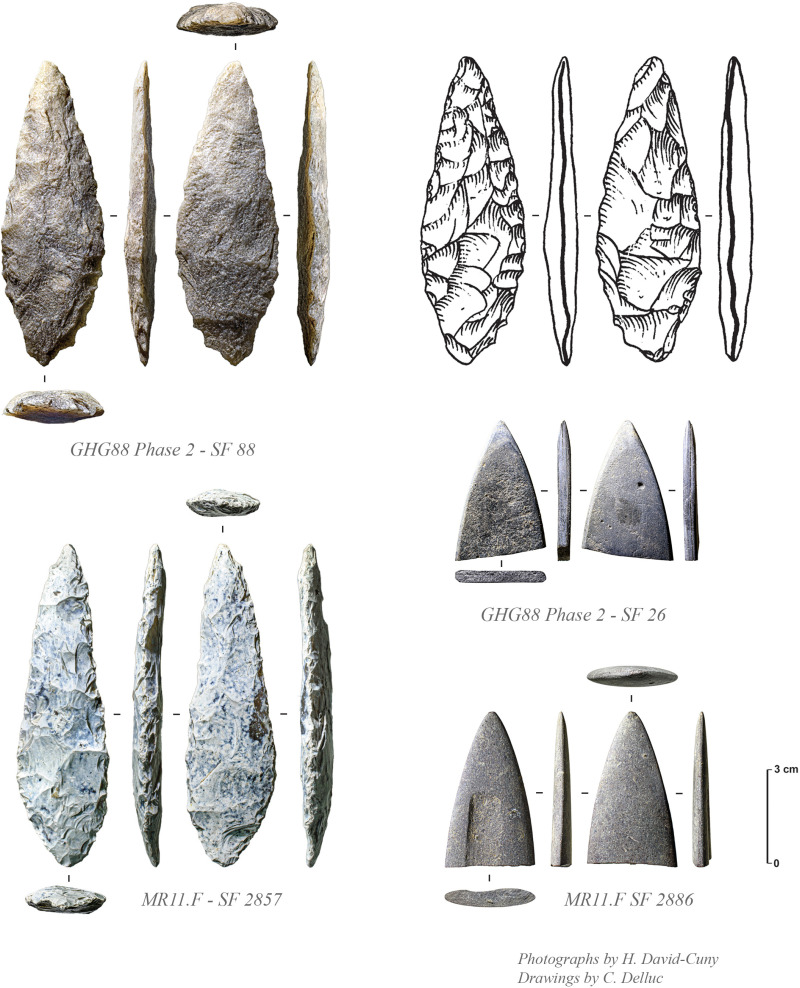
Quartzite ‘spearhead’ (SF88) and polished stone point (SF26) from GHG0088 Phase 2, with comparable specimens from MR11 (Photos: H. David-Cuny; Drawings: C. Delluc).

There are 18 tools in chert, mostly consisting of projectile points (Fig 10), a tile knife and a few bifacial pieces (Fig 11). The assemblage also includes a net sinker with large transversal notches (SF0084). Only two retouched tools are made in quartzite, including a large projectile point (spearhead) (Fig 12, SF0068). While quartz was deliberately knapped, there is no direct evidence of its use for making tools at the site. Debitage is characterised by a limited collection of only 67 complete and fragmentary unretouched flakes, one unretouched blade, and 23 undetermined chunks. Flakes are of small size (mainly 2–3 cm), most of the time being very thin. This is most probably a clear indication of thinning activities made on fine and thin bifacial pieces.

Three small hammerstones can be mentioned as well. One is of a reddish quartz pebble with distinctive striking marks (31x29x19 mm) (SF0222); another one is of a metamorphic rock pebble (quartz mica schist with a foliated structure) (48x34x25 mm) (SF0229). Both are associated with Phase 1a (SU1034 and SU1038). Another small quartzite hammerstone (45x41x24 mm) (SF0013) is associated with Phase 3.

The projectile points at GHG0088 are all characterised by bifacial shaping with symmetric biconvex section (Fig 10). Some of these points are barbed and tanged or of distinctive types, ranging from short to long, with varying measurements of wings and tangs (Table 2). The exclusive presence of flat bifacial points, and the absence of trihedral types, appear as a chronological feature already observed for the late 7^th^ millennium BCE at GHG0014. The presence of complete and fragmentary bifacial pieces made of allochthonous beige fine-grained quartzite is very peculiar and was also already observed at GHG0014 (while absent at MR11). Only one tile knife is documented (Fig 11, SF0002), untypical to the more commonly found forms from MR11 on Marawah Island [20].

**Table 2:** Quantification of retouched lithic tools from GHG0088 assemblage.

Typology	N	Special Finds
Tile knife	**1**	SF0002
Foliated bifacial point	**1**	SF0190
Bifacial short point with long tang and short wings	**1**	SF0242
Bifacial piece	**5**	SF0052; SF0066; SF0068; SF0220; SF0269
Bifacial triangle point with short tang and long wings	**1**	SF0199
Long bifacial point w/ long tang and short wings	**5**	SF0001; SF0102; SF0192; SF0237; SF0271
Net sinker	**1**	SF0084
Retouched blade/flake	**4**	SF0100; SF0126; SF0218; SF0256

The absence of a trihedral tradition at GHG0088, coupled with the exclusive presence of flat bifacial barbed and tanged points, as well as other bifacial tools, underscores a significant shift in lithic traditions between the mid-7^th^ millennium BCE (as seen at GHG0014, GHG0063, and GHG0088) and the mid-6^th^ millennium BCE (MR11). This observation supports the idea that flat bifacial barbed and tanged points were established earlier, as they appear as the sole type of projectile point unassociated with trihedral points. However, during the second main phase of occupation at GHG0088 (Phase 2), which coincides with MR11 occupation, no trihedral points were found. This evidence may suggest a limitation to their spatial distribution, indicating that they did not extend to Ghagha Island [21]. This interpretation is further supported by the lithic assemblage from Shagra (South-East Qatar) [22], located about 50 km northwest of Ghagha Island and dating to the first half of the 6^th^ millennium BCE, where barbed and tanged points are the only type present. Overall, this indicates that lithic traditions in the region were diverse and complex, with potential overlaps, rather than representing a straightforward succession.

#### Groundstone tools.

A flat ogive-shaped point made of polished grey aphanitic (fine-grained) rock (Fig 12 SF0026) – possibly slatestone – was discovered in SU1003 (Phase 2). It measures 4.5 cm in length and 0.4 cm in thickness, with fine-grained polishing evident across its entire surface, resulting in a strict symmetrical profile. Similar objects have been documented in contexts dating back to 5700–5600 BCE at MR11 (Fig 12, MR11.F SF2886), which is consistent with Phase 2 dating at GHG0088.

Other groundstone implements include a large limestone cleaver (SF0047 from SU1005, Phase 2), roughly rectangular and measuring 20x12.5x4 cm. Both ends of the object are bifacially shaped and used. The tool is thick, heavy, and dull. However, it could have been used to break tough materials such as bones or mollusc shells.

Several fragments of millstones were also discovered at the site. The specimens from the lower stratigraphy (Phase 1a) are crafted from a fine-grained reddish sandstone. Two larger specimens (SF0069 and SF0159) found in the upper stratigraphy (Phase 2) are made of fossiliferous limestone – SF0069 was resting on the occupational floor SU1004. Its lower side exhibits distinct smoothing and flattening, indicating prolonged use over time. The surface of SU1004 was covered with numerous tiny fragments of fish bones, suggesting the preparation and consumption of dried fish-derived products, possibly involving their grinding. A comparable millstone was previously found at Khor F.B. (North-East Qatar), a site dating from the first half of the 5^th^ millennium BCE [22].

Other notable finds include irregular stones that appear to have been used as mortar for crushing ochre (e.g., SF0226 from SU1038). They typically exhibit a shallow depression at the centre, with ochre residues. Four specimens were discovered in levels attributed to Phase 1a. These mortars can be compared to the hand-stone (‘mano’) or pestle found in Cell 4 at GHG0014, on which traces of ochre were also identified [4]. The site of Shagra also yielded groundstone tools associated with ochre processing [22]

**Fig 13:**
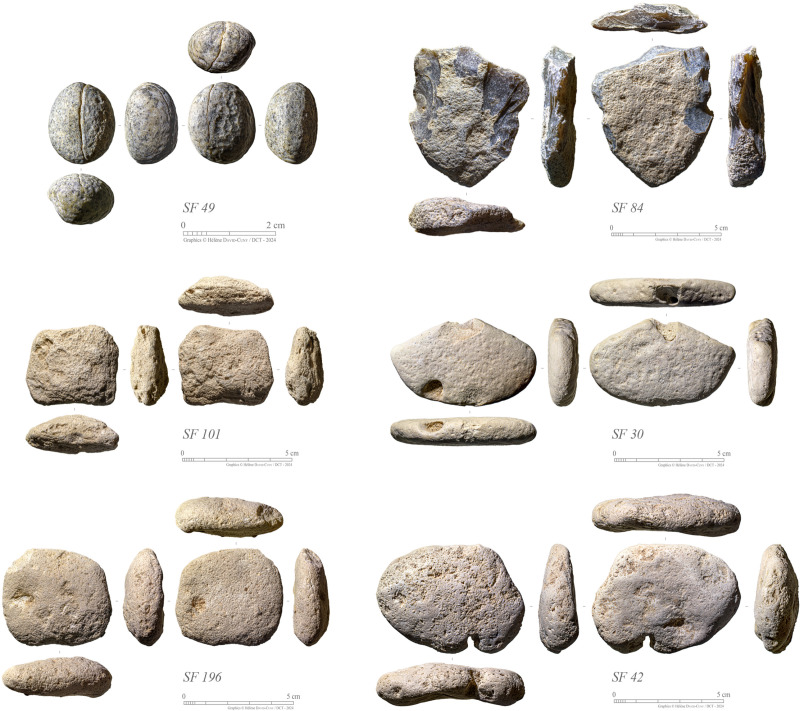
Selection of stone fishing weights from GHG0088 Phase 2 (Photos: H. David-Cuny).

A small assemblage of stone net sinkers has been discovered at GHG0088 (Fig 13). The majority of these sinkers are made from littoral limestone pebbles, totalling fifteen specimens. Half of the sinkers (N = 7) are made of fine-grained micritic limestone, while the other half (N = 8) are made of fossiliferous limestone; both sourced locally. Complete specimens measure up to 7.5 cm in length, 5.5 cm in width, and have a thickness of 2 cm. Notches were created through direct hard-hammer percussion near the middle of the longest sides. The percussion exhibits a high-angle (or abrupt) trajectory, resulting in short removals and crush marks. These sinkers align typologically with Type 1.B as classified by Lidour (2023). Additionally, a unique specimen crafted from a knapped chert pebble (SF0084) from SU1006 displays significant removal negatives from its longest sides, and it is categorised as the ‘Clactonian’ variant of Type 1.B, as also documented in the Sultanate of Oman: by Uerpmann [23] at Khor Milkh 1 (4^th^ millennium BCE) and Charpentier et al. [24] in levels dating to the end of the 4^th^ millennium BCE at Suwayh 2.

It is noteworthy to mention the presence of a singular line sinker (SF0049 from SU1005), made from a small igneous rock (plutonic rock) pebble. It is roughly oval-shaped and measures 1.8x1.5x1.6 cm. It bears a thin (approximately 0.6 mm) and continuous incised line encircling its longest waist. The groove exhibits a slightly bevelled V-profile in its better-preserved parts. Miniature line sinkers with an incised line are linked to Type 2.B from Lidour [2]. They were probably utilised for weighting luring lines with shell fishhooks during the latter part of the Neolithic period in the Sultanate of Oman and the UAE. Interestingly, the only other specimen of this type found in the UAE is associated with the mid-4^th^ millennium ceremonial dugong structure at Akab (Emirate of Umm al-Quwain) [25,26]. Other specimens from the Sultanate of Oman (Saruq in the Muscat area, Ras Al Hamra RH6, and Ras Al Khabbah KHB1) further support the hypothesis that these artefacts are closely linked to contexts of the latter half of the 4^th^ millennium BCE [23,27,28]. However, SF0049 is associated with Phase 2, which has been dated from the first half of the 6^th^ millennium BCE, suggesting an earlier date for this type of fishing weight.

All the fishing sinkers are associated with Phase 2, and the majority of specimens were retrieved from SU1004. This highlights the importance of fishing activities at the site.

#### Shell tool assemblage.

**Fig 14:**
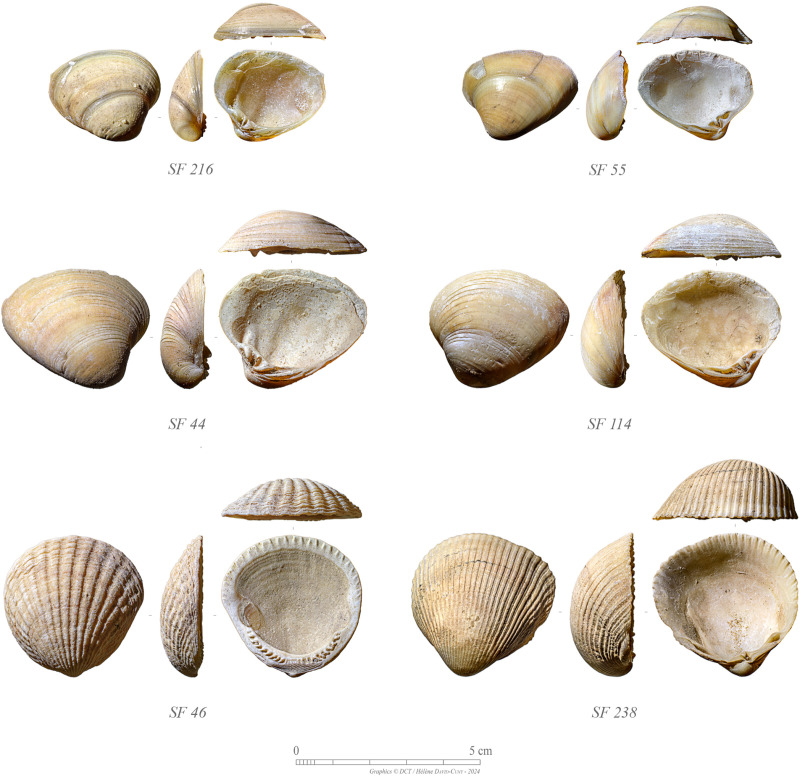
Selection of marine shell tools from GHG0088: shell scrapers or knives made from *Callista umbonella* valves (SF216, SF55, SF44, and SF114); possible shell containers made from *Glycymeris* sp. (SF46) and Fulvia fragilis (SF238) valves (Photos: H. David-Cuny).

A relatively important collection of shell tools was unearthed during the excavations considering the small size of the site (Fig 14). A total of 13 retouched complete valves and 21 valve fragments of *Callista umbonella* were found at GHG0088. Specimens are reported from both Phase 1 (N = 10) and Phase 2 (N = 24).

The retouch is typically concentrated along the ventral margin of the valve – they were likely made by direct and hard percussion [29,30]. Recent use wear analysis of shell tools from the late 6^th^ millennium BCE site of UAQ2 has revealed their use in a variety of productive activities, including processing vegetal fibres and treating animal skins with ochre – thus used both as scrapers and knives [31]. Similarly, 3 fragments of valve from Phase 1 at GHG0088 show stains of ochre on their inner side, suggesting a similar use. Additionally, a few unworked valves of *Mimachalmys senatoria*, *Spondylus spinosus*, *Glycymeris* sp., and *Vasticardium* sp. from Phase 2, plus a *Fulvia fragilis* valve from Phase 1, seem to have been used and thus have been identified as potential shell tools. Future functional analysis will provide more detailed insights into the use of the earliest shell tools found currently in the Arabian Peninsula: at GHG0088 and at the neighbouring site of GHG0063, both dating to around 6400–6200 BCE.

A total of 11 non-retouched valves of *C. umbonella* were found in contexts associated with Phase 2 as well. These are thought to have been collected as raw material and were intended to be modified into tools when needed.

#### Plaster vessel.

**Fig 15:**
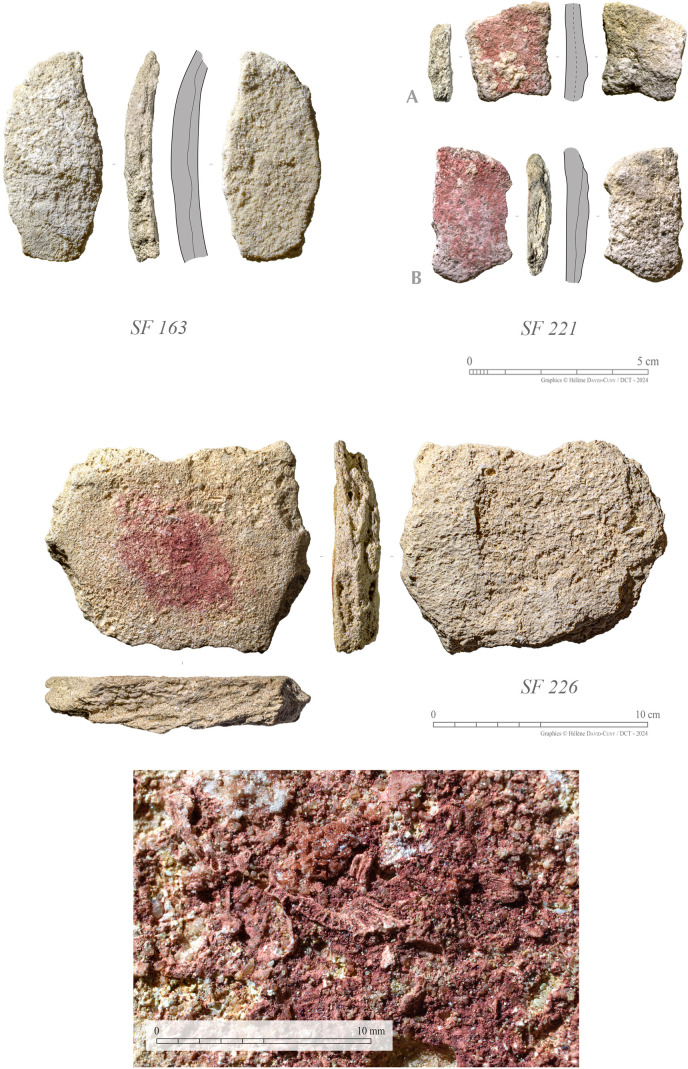
Selection of plaster vessel sherds from GHG0088, including a specimen of local stone used as a mortar for crushing ochre, notably for decorating the plaster vessel (Photos: H. David-Cuny).

Evidence of plaster vessel is limited at the site (Fig 15), gathering a total of 7 sherds only. No fragments of rims or bases were identified. However, the profiles of the sherds suggest these are possibly from an open rounded bowl. Their average thickness is around 6–8 mm. Almost all sherds are associated with Phase 1. A single sherd is associated with Phase 2 (SF0163) – retrieved from SU1014.

The plaster sherds recovered from GHG0088 are homogenous with those recovered from the nearby Neolithic sites on Ghagha Island (GHG0014 and GHG0063) [4]. The fabric is quite fragile and porous and contains frequent inclusions of gypsum crystals and microcharcoals. Plaster wares are typically built up in layers either to reinforce or to repair, as needed. The sherds collected from GHG0088 present a minimum of two layers, with at least one internal surface.

The painting is a faded red pink and is extensively applied on the exterior face of the ware. The pigment is made of a smeared powdered haematite – probably sourced from nearby salt dome formations, such as the ones found at Delma (80 km), Sir Bani Yas (100 km), or Jebel Dhanna (110 km). The decoration was most likely added before the vessels were completely dried. No patterns are visible, as the small size of the sherds limits the degree of visual assessment that can be performed. Painted sherds are present in SU1017 and SU1038 – where large stones have been used for crushing pigments. No traces of incisions or reduction scars are visible on the exterior face of the vessel; these are more commonly observed in later plaster ware decorations. Culturally, these plaster ware sherds represent some of the earliest local wares in the region.

#### Personal adornment.

**Fig 16:**
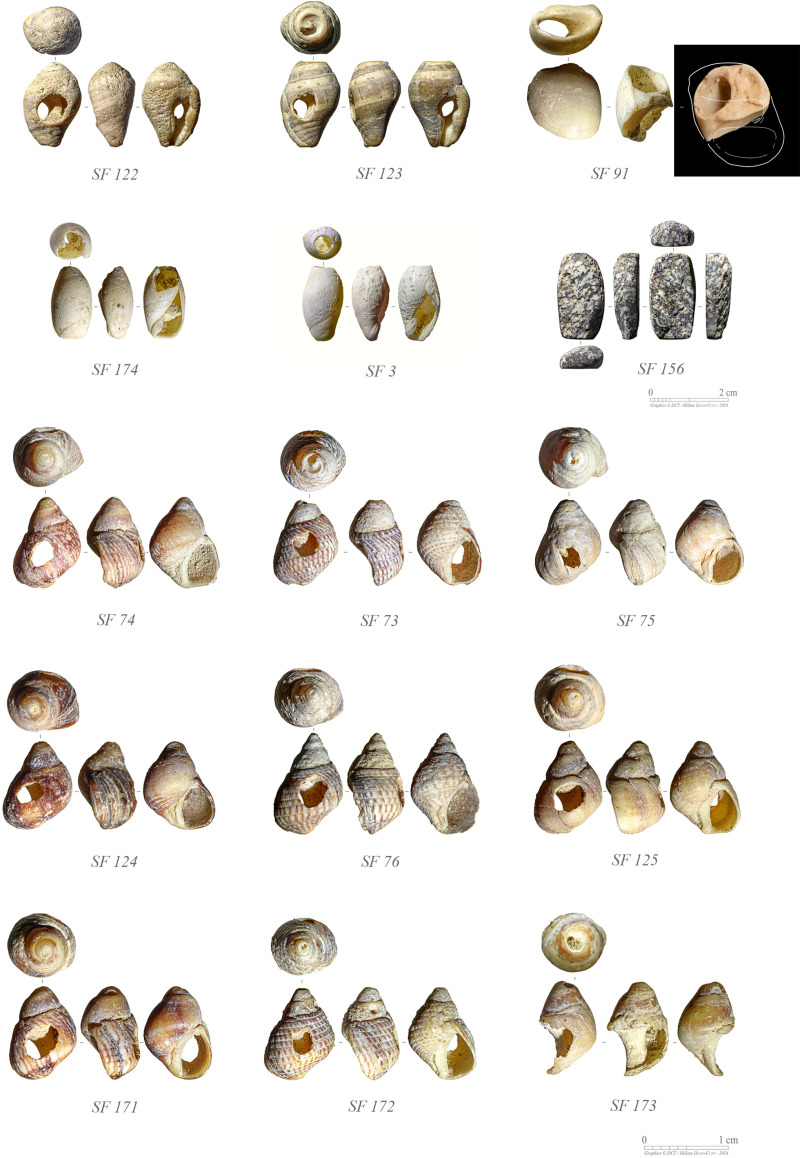
Ornaments from GHG0088: beads made from *Engina mendicaria* (SF122–123), *Polinices mammilla* (SF91), and *Planaxis sulcatus* (SF73–76, SF125, and SF171–173) perforated shells; beads made from *Ancilla farsiana* (SF174, SF3) shells with the apex cut off or abraded; preform of beads or pendants made from igneous rock (SF156) (Photos: H. David-Cuny; Drawings: K. Lidour).

A number of marine shell beads were retrieved from GHG0088 (Fig 16; Table 3). They are all perforated gastropod shells, recovered from the Phase 2 occupation and thus associated with a mid-6^th^ millennium BCE dating. *Planaxis sulcatus* shell beads (N = 9) are locally sourced, with associated mollusc being still present in quantities along the surrounding shores. Two *Engina mendicaria* and a *Polinices mammilla* shell beads were imported. These species are not encountered on Ghagha Island, or the southern Arabian Gulf. According to Bosch et al. [32], living specimens are recorded in the Gulf of Oman and the Arabian Sea, evidencing long-distance interaction. An important workshop of *E. mendicaria* beads is documented at Al Haddah BJD1 (close to Ruways, in the Sultanate of Oman) [33]. They were also extensively exchanged across the Arabian Gulf during the Neolithic (e.g., c. 4900–4700 BCE at Dosariyah, in Saudi Arabia) and the Bronze Age, up to Southern Mesopotamia (modern Iraq) – in the royal tombs of Ur (Tell el-Muqayyar) and Kish (Tell al-Uhaymir) during the 3^rd^ millennium BCE [34,35].

**Table 3:** General table of artefacts by phase. P.3* also includes deflated levels and thus might provide both Neolithic and Islamic artefacts.

			P.1a	P.1b	P.2	P.3*	Total
Lithic industry	*Chert-Flint*	Projectile point	2	–	1	5	**8**
		Projectile point on retouched blade?	1	–	–	–	**1**
		Projectile on bifacial piece?	–	–	–	1	**1**
		Bifacial piece	–	–	2	2	**4**
		Tile knife	–	–	–	1	**1**
		Retouched flake	–	–	2	–	**2**
		Debitage	3	–	23	22	**48**
	*Quartzite*	Projectile point (spearhead)	–	–	1	–	**1**
		Retouched flake	–	–	–	1	**1**
		Debitage	1	1	11	11	**24**
	*Quartz*	Pebble	–	–	34	7	**41**
		Debitage	2	–	19	–	**21**
	*Others*	Polished stone point	–	–	1	–	**1**
		Peridotite pebble	–	–	–	1	**1**
		Black stone fragment	4	1	39	24	**68**
		Red jasper pebble	–	–	–	1	**1**
		Granite pebble	–	–	–	1	**1**
		Rhyolite fragment	–	–	–	1	**1**
Groundstone		Net sinker	–	–	15	2	**17**
		Line sinker	–	–	1	–	**1**
		Hammerstone	2	–	–	1	**3**
		Millstone	2	–	3	–	**5**
		Anvil	1	–	–	–	**1**
		Ochre mortar	4	–	–	–	**4**
		Limestone cleaver	–	–	1	–	**1**
Shell tool kit		*Callista umbonella* valve (retouched)	8	1	26	3	**38**
		*Callista umbonella* valve (non-retouched)	–	–	11	–	**11**
		*Mimachlamys senatoria* valve	–	–	2	2	**4**
		*Fulvia fragilis* valve	–	–	–	1	**1**
		*Glycymeris* sp. valve	–	–	1	–	**1**
		*Spondylus spinosus* valve	–	–	1	–	**1**
		*Vasticardium* sp. valve	1	–	–	–	**1**
Plaster vessel		Sherd	6	–	1	–	**7**
Pottery		Julfar ware sherd	–	–	–	9	**9**
Personal adornment	*Granite*	Pendant preform	–	–	1	–	**1**
	*Marine shell*	*Ancilla farsiana* bead	–	–	1	1	**2**
		*Engina mendicaria* bead	–	–	2	–	**2**
		*Nassarius* sp. bead	–	–	2	–	**2**
		*Polinices mammilla* bead	–	–	1	–	**1**
		*Planaxis sulcatus* bead	–	–	9	–	**9**
		Cowrie shell	–	–	2	–	**2**
Miscellaneous	*Glass*	Glass bangle fragment	–	–	–	3	**3**
		**Grand total**	**37**	**3**	**213**	**100**	**353**

Both *P. sulcatus* and *E. mendicaria* shells were perforated by hard-hammer percussion on the dorsal side of their body whorl. For *P. mammilla*, the perforation is made following the same technique but on the ventral side of the body whorl. Two further shell beads are made from small *Ancilla* cf. *farsiana* with the apex cut off (or abraded). These shells could have been locally obtained. A unique cowrie shell (SF0070) was found associated with SU1004 from Phase 2 as well.

A fragment of plutonic rock pebble (SF0156 from SU1009) was polished on most of its surface. It shows a roughly rectangular shape, suggesting it might represent a preform for some kind of bead or pendant.

## Discussion

### Chronology of the early Arabian Neolithic

Charcoal fragments were found in contexts associated with Phase 1a, allowing for the absolute dating of the occupation to around 6400–6200 BCE. Phase 1a at GHG0088 is thus contemporaneous with the neighbouring site of GHG0063 and at least 100 years younger than GHG0014 [4] (Table 4), where another stone-built structures were investigated. Apart from the debitage products (chert, quartz, and quartzite) that are present throughout the site’s occupation, some differences in the material culture highlight chrono-cultural changes between the different phases. Firstly, the projectile points are more prevalent during Phase 1a, with some specimens occurring in deflated deposits around the main excavation area. These projectile points are likely associated with the oldest occupation on the site. The projectile points found in Phase 1a are barbed and tanged, commonly documented on other Neolithic settlements on Ghagha Island (i.e., GHG0014, GHG0063) [20].

**Table 4:** Tentative chronology for the Early Neolithic period in the Arabian Gulf based on the comparison of sites from Ghagha and Marawah.

Cultural period	GHG0088 phasing	Age range	Compared site
Early Arabian Neolithic 1	Phase 1a	6600–6200 BCE	GHG0014, GHG0063
	Phase 1b	6200–5700 BCE	
Early Arabian Neolithic 2	Phase 2	5700–5400 BCE	MR11

The excavations at GHG0088 yielded a limited number of artefacts, suggesting that the site may not have been occupied for extended periods. Most of the finds are concentrated in Phase 2 (213 artefacts out of 353, c. 60%) (Table 3), which dates to 5700–5400 BCE. The absence of Mesopotamian pottery (Samarra-Ubaid tradition) on-site also supports this dating since there is no evidence for its diffusion in the Arabian Gulf before 5550 BCE [36,37]. A single fragment of a projectile point made from chert (SF0102) was discovered in association with the Phase 2 occupation. It appears to be the tang of a bifacial point. The fragment has a biconvex, asymmetrical cross-section and does not belong to a trihedral type. Trihedral points are associated with the Middle Neolithic (c. 5500–4500 BCE) in the Sultanate of Oman according to the Suwayh SWY1 chronology [38–40]. The discovery of specimens associated with contexts dating back to around 5800–5600 BCE at MR11 suggests a revision of their chronology, placing their existence slightly earlier than previously believed [41]. Despite the absence of trihedral points at GHG0088, a single elongated quartzite point (Fig 12 SF0068) was found in Phase 2 – the latter is typologically comparable to a chert ‘spearhead’ found in 2022 in the MR11 Area F Burial 1115 (Fig 12 SF2857), also dated around c. 5700–5600 BCE [4].

The thick aeolian sand deposit between Phase 1 and Phase 2 accumulated from 6200 BCE onwards, according to the results from radiocarbon dating. At GHG0014, a comparable 50 cm thick layer of aeolian sand sealed the occupational layers dated from 6600–6500 BCE. An Early Bronze Age burial dated to around 2900–2700 BCE was installed atop this sand accumulation [4]. The end of the domestic occupation of both GHG0014 and GHG0088 Phase 1a by the end of the 7^th^ millennium may be attributed to the onset of a more arid climate, likely associated with the 8.2 ka BP event (Bond Event 5) [42]. Paleoclimatic records from speleothems sampled from the Sultanate of Oman have indicated reduced rainfall from 8.2–8.0 ka BP, associated with a weakening of the Indian Ocean Monsoon system [43,44]. Reduced rainfall may have impacted the availability of local resources and forced coastal foragers to relocate their habitat, or even to adopt a more nomadic lifestyle. Nevertheless, paleoclimatic data from Fleitmann et al. [44] also indicates a short-lived recovery in monsoon intensity after the 8.2 ka BP event, peaking between 5900 and 5600 BCE, before a long-term trend that continued towards decreasing precipitation. In this sense, Phase 1b at GHG0088 has provided evidence of human activity about 6000–5800 BCE. The re-occupation of GHG0088 during Phase 2, by 5700–5400 BCE, seems associated with the development of the MR11 site.

Freshwater availability at coastal sites during the Arabian Neolithic remains underexplored. Evidence from UAQ2 indicates that animal milk was utilised to mitigate water scarcity since the late 6^th^ millennium BCE [45]. Early Neolithic sites, such as on Ghagha and Marawah Island, however, lack clear evidence of herding, implying domesticated animals may have arrived later in the region. During the Early Holocene, increased rainfall likely improved water availability from springs, streams, and catchment systems [6,42]. Groundwater recharge models for the Liwa oasis in the UAE estimate an annual precipitation of 200 ± 50 mm/yr for 9.0–6.2 ka [46], approximately 4–5 times the current levels.

The definition and chronology of the Neolithic period in the Arabian Peninsula remain highly debated, primarily among a small group of scholars specialising in the region. Recent chronologies have often relied on lithic facies, particularly projectile points [23,38,40], which may introduce a bias regarding the diversity of material cultures. Notably, most assemblages used to develop these chronologies originate from the southern Sultanate of Oman. For this study, we propose a revised chronology of the Arabian Neolithic from a sub-regional perspective, allowing for a focus on more comparable sites within a smaller geographical framework.

Sites from Ghagha Island represent the oldest Holocene stratified sites in the Arabian Gulf, forming the basis for defining an Early Arabian Neolithic. The internal division of this period and the transition to a Middle Arabian Neolithic are directly based on the GHG0088 phasing, with MR11 recognised as the major site for an Early Arabian Neolithic 2. The start of the Middle Arabian Neolithic is placed in the mid-6^th^ millennium BCE, marked by the diffusion of Mesopotamian pottery in the Gulf. DLM0019 and UAQ2 are considered key sites from this period in the UAE, alongside As-Sabiyah H3 (Kuwait) and Dosariyah (Saudi Arabia) from other parts of the Arabian Gulf [34,47]. The Late Arabian Neolithic is less documented in the Gulf, primarily represented by the site of Akab, inhabited from the mid-5^th^ millennium BCE.

### Neolithic stone-built architecture

The location and construction of these stone structures reflect an adaptation to the local environment. The site’s topography provides a vantage point that overlooks the neighbouring sea and facilitates access to marine resources. This likely influenced the choice to locate the structure. The state of the exposed ground surface is also to be considered. The architecture is built at the base of a small limestone ledge, which has been likely purposed as a natural wall – further elevated by stone courses. This natural feature is likely to have been originally used as a windbreak against the prevailing NNW winds blowing into the Arabian Gulf region. An initial phase of occupation could have taken place prior to the construction of any coursed stone structure; however, this has not been preserved in the stratigraphy. The bedrock on GHG0088 is uneven, but lower stone courses frequently show strategic placements that contribute to the general stability of the structure.

The GHG0014 structure is more sophisticated in design (six cells) and better preserved (walls up to 1 m high, 12 courses of stones) than at GHG0088. However, the GHG0088 main cell includes complex internal features that are likely associated with later additions and modifications of the original plan. Both of these sites share a number of similarities in terms of architecture: 1) The circular-cell ground plan; 2) The use of unworked local limestone to build the walls, with flatter stones placed strategically in the courses; 3) The walls from GHG0088 and GHG0014 are of a similar thickness, roughly half a metre in width (50–60 cm). These characteristics are also reminiscent of later structures found on Marawah Island (in particular at MR11). Another feature that is more comparable to MR11 is the use of smaller cells for funerary purposes – which has not been observed at GHG0014.

The roof was likely made of wood branches and other vegetal materials, including shrubs and thickets. At MR11, there is evidence of large limestone slabs being used to weigh down and block a light roofing [48]. These slabs are often found vertically and obliquely standing in the rubble deposits, suggesting that they fell into the rooms as the vegetal roof decayed. Similar findings have not been made at GHG0014 or GHG0088. According to Al Hameli et al. [4], at GHG0014, natural dips in the bedrock may have helped to stabilise wooden posts aimed at holding up the roof. Some of the internal features observed in the GHG0088 main cell may have helped prevent hypothetical posts from slipping out of their foundation. GHG0088 thus appears as a transitional example in the development of the Early Neolithic stone-built architecture in the region by associating elements of the Ghagha and Marawah traditions.

Similarities can also be drawn with the site of Shagra in southeastern Qatar, where a 3x5 m oval stone structure has been documented [22]. It dates to the first half of the 6^th^ millennium BCE, making it roughly contemporaneous with GHG0088 Phase 2 or the MR11 structures. The Shagra structure features an internal compartment and a 1x1 m appendix in its southwestern wall. The entrance has been identified along the southeastern wall, strategically protected from prevailing winds. Unlike the structures on Ghagha and Marawah, the outline of the Shagra structure is not composed of stone courses forming walls but rather of rows of orthostats, which are thought to have supported vegetal walls (a hypothesis confirmed by pollen analysis [49] conducted inside the structure).

In this respect, the Shagra structure differs from what is observed on Ghagha and Marawah, with the exception of Room 8 in MR11 Area C, which is roughly circular (approximately 2.5 m in diameter) and notably contained human remains [41]. However, evidence of domestic activities at Shagra—such as lithic knapping (nearly 2400 flakes), the use of groundstone, and fish consumption—challenges the idea of a funerary purpose for the structure, at least for the part that has been excavated.

### Evidence of a human burial?

One significant finding related to GHG0088 is the presence of human bones during Phase 1b in the small cell, raising questions about its funerary purpose. The small size of the internal space suggests its use as a burial chamber, which is supported by the results of excavations on Marawah Island. Indeed, recent excavation at MR11 in 2022 have provided a detailed understanding of the architectural organisation, revealing large domestic and communal rooms flanked by smaller cells used as burial chambers [50].

Similarly, at GHG0088, the main cell appears to have been associated with domestic activities, indicated by the presence of lithic industry and faunal remains. Although potential human bones were discovered inside the small cell, their absence in the lower deposits directly following construction seems to reject the hypothesis of the disturbance of a primary burial. However, due to the lack of information on preservation conditions over almost nine millennia of depositional activity on site, this cannot be stated firmly.

### Early maritime adaptations

According to archaeological evidence, maritime adaptations appear to be a relatively recent cultural development in the Arabian Peninsula. An early fishing-based economy emerged around 8500 BCE at the Natif 2 HBM-10 cave (Dhofar region, Sultanate of Oman), demonstrating a strong reliance on the exploitation of marine molluscs and coastal fish [51,52]. This timeline is supported by the discovery of two sea snail shells (Turbinidae), which are also dated by 8500 BCE, in the upper part of the stratigraphy of the Jebel Faya FAY-NE1 rock shelter (Emirate of Sharjah, UAE) [53], indicating contact with the coast located 50 km away. These findings align with the conclusion of the Terminal Pleistocene hyperarid phase and the beginning of wetter conditions associated with the northward migration of the Intertropical Convergence Zone (ITCZ) [54]. In the context of the postglacial marine incursion, newly formed shallow sea basins reached by either intermittent or perennial watercourses created favourable conditions for the gradual establishment of tropical coastal ecosystems. This includes mangroves, seagrass meadows, and coral reefs, which are recognised as significant marine ‘biodiversity hotspots’ [1]. GHG0014 is the oldest yet documented coastal Neolithic settlement site in the Arabian Gulf, dated to 6600–6500 BCE. It has a strictly fishing-based economy, as no remains associated with the consumption of terrestrial mammals were found [4]. The fishing strategy is overall based on the exploitation of coastal fish, with the virtual absence of molluscs. By contrast, small quantities of molluscs are present from c. 6400–6200 BCE at both GHG0063 and GHG0088. These molluscs primarily consist of *C. umbonella* valves that have been reused as shell tools. They were likely locally collected on the shore. More recent occupations on Ghagha Island have exploited the abundance of certain species of edible molluscs in the surrounding waters, in particular, murex species (e.g., *H. kuesterianus*) and Gulf pearl oysters (*P. radiata*).

Similarly to GHG0014, the virtual absence of marine mollusc shells in Phase 1 could be due to the young age and the shoreline instability of the Arabian Gulf by the 7^th^ millennium BCE. During the Late Glacial Maximum (20–15 ka), the Arabian Gulf underwent a significant retreat caused by a global sea level drop of c. 120m. The subsequent post-glacial eustatic rise reactivated the filling of the Arabian Gulf [55,56], and by around 8000 BCE, it was thought to have been one-third full, with shorelines continuing to advance rapidly until 4000 BCE. The colonisation of new habitats by marine molluscs can take several decades – or centuries – depending on species and environmental conditions. Bivalves and gastropods rely on larval dispersal, which is influenced by currents, temperature, and suitable substrates. In contrast, fish species are expected to colonise new areas more quickly due to their greater mobility and ability to cover larger distances [57,58]. Under favourable conditions, certain fish species can establish new populations within a few years.

The presence of stone net weights during the second phase of occupation at the site underscores the importance of fishing activities. This supports the idea that the site was most likely reoccupied by ancient fishers. This community probably utilised the locally available stones from the collapsed architecture to establish a camp—raising the question of whether the occupation was temporary or permanent in nature. The total amount of fish remains collected is limited in Phase 2, which could be linked to the poor preservation of the bones in the uppermost deposits of the site. This could also be a result of the small size of the fish captured, such as sardines, anchovies or juveniles. The apparent specialisation in the capture of small fish during Phase 2 clearly differs from the prevalence of medium-sized groupers and bottom-feeder fish during Phase 1. Large and slow swimming fish are expected to have been captured using spears or harpoons in shallow waters – tools that might have implied the use of some of the lithic projectile points found at the site. However, nothing avoids a concomitant and complementary use of fishing nets and spears in the fishing effort. It might have been the case at MR11 as well. However, the increased variety of fish catches at MR11 is attributed to the more diverse marine habitats around Marawah Island compared to Ghagha Island, rather than to a change of the techno-cultural pattern through time [59,60].

## Conclusion

Recent archaeological discoveries on Ghagha Island provide a valuable lens through which the unique trajectory of neolithisation in the Arabian Gulf can be studied. The absence of herding and agriculture, coupled with the strong reliance on marine resources, challenges traditional definitions of the Neolithic and underscores the importance of local environmental conditions in shaping cultural evolution.

The site of GHG0088 has revealed two main phases of occupation, shedding light on the evolution of regional architecture, material culture, and subsistence strategies. The following chronology is suggested in the study context of the Abu Dhabi islands. It aims to highlight the sub-regional variations within the Arabian Neolithic. The earlier phase (6400–6200 BCE), identified as belonging to the Early Arabian Neolithic 1 (‘Ghagha Phase’), is marked by the construction of a circular stone-built structure that are associated with a lithic industry, shell tools, and painted plaster ware. A smaller cell added around 6000–5800 BCE, containing human remains, suggests funerary practices similar to those at Marawah Island (5800–5600 BCE). GHG0088 thus represents a transitional stage in the development of Neolithic stone architecture in South-East Arabia, by exhibiting a unique combination of building traditions seen at Ghagha and Marawah. The second phase, dating to the first half of the 6^th^ millennium BCE, highlights the site’s reuse by a fishing community, evidenced by stone net weights. The material culture of this phase shows several similarities with examples from MR11 and thus constitutes an important comparative assemblage for the Early Arabian Neolithic 2 (‘Marawah Phase’) in the Abu Dhabi islands.

Similar to the other Neolithic sites on Ghagha Island, the faunal assemblage at GHG0088 is exclusively marine. The virtual absence of terrestrial fauna in the Abu Dhabi islands (i.e., Ghagha, Delma, Marawah) during the Neolithic contrasts with contemporaneous mainland sites like UAQ2 [45], hinting at divergent economic strategies between insular and continental communities. Nevertheless, the presence of shell beads that are thought to originate from the Gulf of Oman, highlighting long-distance exchange. Early coastal adaptations include the production and use of retouched shell tools from 6400–6200 BCE at both GHG0088 and GHG0063, while totally absent at GHG0014, only one or two centuries earlier.

Further comparative studies between GHG0088, GHG0063, and GHG0014 will undoubtedly enhance our understanding of the sequence of occupation on Ghagha Island and allowing for insights into the cultural and economic dynamics of the Early Arabian Neolithic in South-East Arabia. This research will provide a broader perspective on human adaptations to coastal environments during the first half of the Holocene, a crucial period in human history. The Early Holocene brought significant environmental changes, including the development of rich coastal ecosystems that acted as ecological refuges and catalysts for cultural innovation. These changes likely triggered a “cultural revolution,” fostering the emergence of stone-built architecture, plaster vessel production, and long-distance exchange networks. Despite the Arabian Gulf’s relative youth at the end of the 7^th^ millennium BCE, marine resources are expected to have been sufficient and perennial all year round to support low residential mobility and an early form of sedentarism [61]. These findings directly align with Desse’s preliminary assessments regarding the seasonality of the Shagra site, occupied during the early 6^th^ millennium BCE [62].

The findings at GHG0088 also further affirm that the neolithisation process in South-East Arabia was deeply rooted in marine adaptations [2], distinct from the agrarian-based traditions of neighbouring regions. The lack of data for the Upper/Late Palaeolithic in the Arabian Peninsula leaves critical gaps in understanding the precursors to the Neolithic, highlighting the need for further exploration of earlier periods to fully understand the unique pathways of neolithisation in South-East Arabia.

## Supporting information

S1 File 3D rotational view of the archaeological site GHG0088 (Phase 1), generated using Agisoft Metashape Professional 2.1.0. The video file is available on Figshare under a CC BY 4.0 license, https://doi.org/10.6084/m9.figshare.28564031.v1.
(URL)

S1 Fig Stratigraphic matrix of GHG0088, processed using Le Stratifiant 0.3.7 (Microsoft Excel macro commands program by Desachy 2008). (JPG)
