# Supplementary figures and images for: Exploring the Early Neolithic in the Arabian Gulf: A newly discovered 8,400–year-old stone-built architecture on Ghagha Island, United Arab Emirates
Source: PLoS One. 2025 Jun 25;20(6):e0326259. doi: 10.1371/journal.pone.0326259 (PMC12194024; doi:10.1371/journal.pone.0326259)

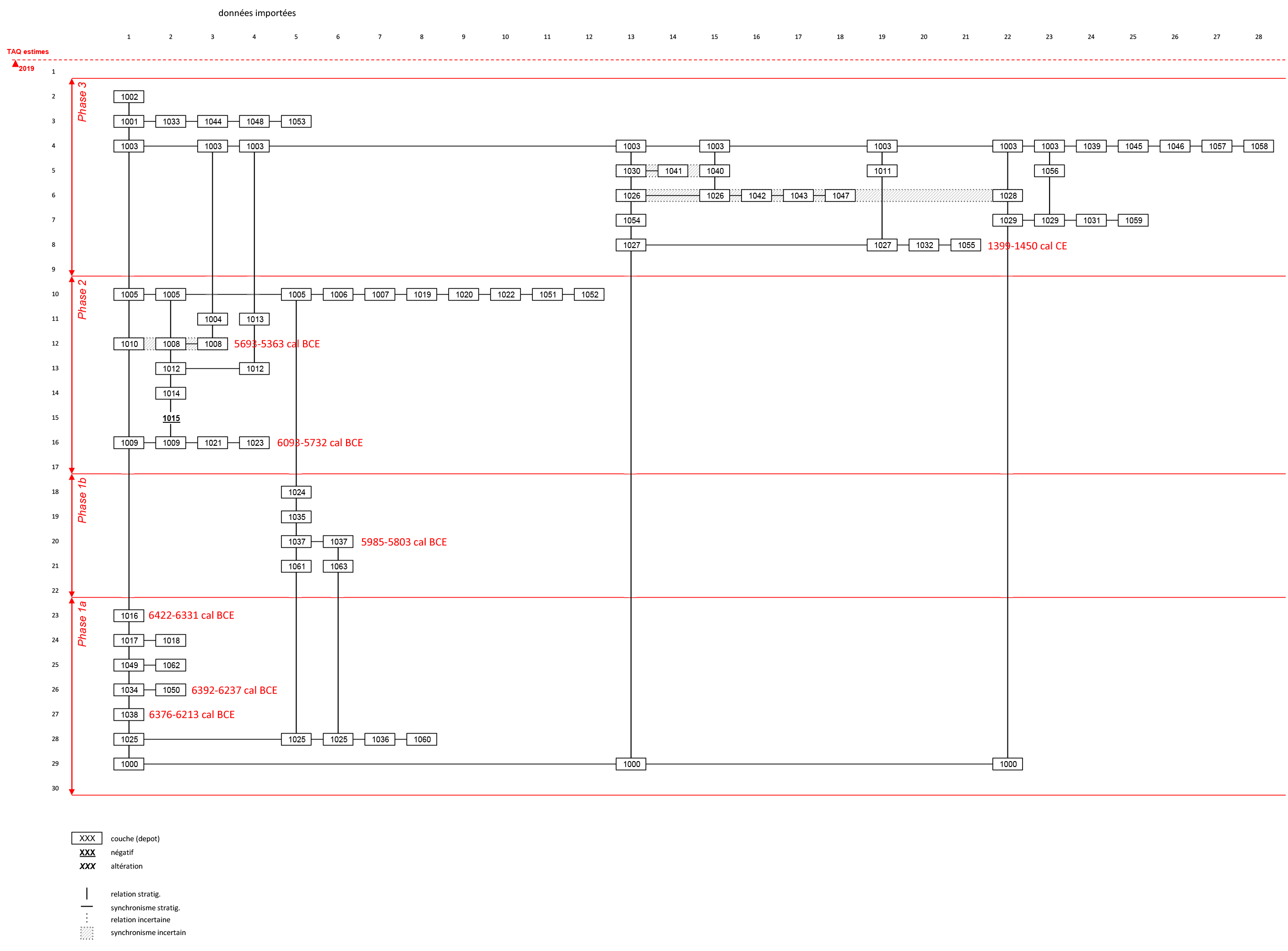

Supplement: S1 Fig — (JPG) [file pone.0326259.s002.jpg]
